# Validity and reliability of running gait measurement with the ViMove2 system

**DOI:** 10.1371/journal.pone.0312952

**Published:** 2024-10-31

**Authors:** Rachel Mason, Gillian Barry, Gary Hall, Alan Godfrey, Samuel Stuart

**Affiliations:** 1 Department of Sport, Exercise and Rehabilitation, Northumbria University, Newcastle upon Tyne, United Kingdom; 2 Department of Computer and Information Sciences, Northumbria University, Newcastle upon Tyne, United Kingdom; 3 Department of Neurology, Oregon Health and Science University, Portland, Oregon, United States of America; Aichi Prefectural Mikawa Aoitori Medical and Rehabilitation Center for Developmental Disabilities, JAPAN

## Abstract

Running biomechanics have traditionally been analysed in laboratory settings, but this may not reflect natural running gait. Wearable technology has the potential to enable precise monitoring of running gait beyond the laboratory. This study aimed to evaluate the analytical validity and intra-session reliability of temporal running gait outcomes measured by the ViMove2 wearable system in healthy adults. Seventy-four healthy adults (43 males, 31 females, aged 18–55 years) wore the inertial device, ViMove2 on the tibia. Participants ran on a treadmill for one minute at various speeds (8, 10, 12, 14km/hr), completed in a standardised shoe (Saucony Guide Runner). Running gait was measured with the ViMove2 wearable and 3D motion capture (Vicon). Temporal running gait outcomes included ground contact time (GCT) and cadence (steps/min). GCT and cadence from the ViMove2 had face validity with expected changes in outcome with different running speeds, but ViMove2 tended to over-estimate GCT, and under-estimate cadence compared to the reference, especially at slower speeds. GCT demonstrated moderate to good agreement to the reference at speeds >10km/hr, but poor agreement at 8km/hr and within female runners. Cadence had moderate to excellent agreement across speeds compared to the reference. GCT and cadence had excellent reliability across speeds, but at 8km/hr GCT had good agreement between trials. Overall, temporal gait outcomes of GCT and cadence can be measured with the ViMove2, but accuracy and reliability are impacted at slow running speeds and within female runners. Future work is needed to clarify sex or speed-dependent corrections to algorithms / outcomes to aid interpretation and application.

## Introduction

Running is one of the most popular sport and recreational activities worldwide, consistently ranking within the top five most favored activities, with participation rates spanning from 7.9 to 13.3% globally across the six regions designated by the World Health Organization (WHO) [1]. Further to this, running is a core component of many sports. The utilization of quantitative running gait analysis, both as a means to reduce injury risks and as a metric for performance evaluation, has received considerable attention in scholarly literature [2–4]. The analysis of running gait carries substantial practical implications for improving athletic performance and assisting in the diagnosis and monitoring of injuries or medical conditions in clinical settings [5–7]. Traditionally, running gait analysis has been conducted in laboratory settings employing diverse methodologies, including 2D video analysis [8, 9], 3D motion-capture systems [10], force plates [11], instrumented walkways or mats, and treadmills [12–14]. Despite their accuracy in measuring gait parameters, these conventional methods pose challenges such as high equipment costs, the necessity for skilled personnel, and the inconvenience of laboratory visits for participants. Moreover, laboratory-based assessments may not authentically replicate real-world running conditions, thereby limiting their applicability in sports and clinical settings [5, 15].

Wearable technology presents a cost-effective and lightweight solution to address the constraints linked with conventional methods of assessing running gait [16]. The increasing acceptance and adoption of wearable technologies among sports practitioners, athletes, patients, and clinicians are notable, with approximately 90% of runners incorporating some type of technology to track their progress [17]. Wearable technology using accelerometers, gyroscopes, and magnetometers, applied individually or in combination as an inertial measurement unit (IMU), allow quantification of running gait outcomes and have become a viable alternative due to their portability and affordability [4, 18, 19]. However, recent research suggests that while wearable technologies for running gait assessment are gaining popularity, less than 10% of commercially available wearables for gait analysis undergo analytical validation against an accepted ’gold-standard’ (reference tool) [20] and even fewer establish the reliability of gait outcomes derived from wearable technology [19]. Ensuring the analytical validity and reliability of these wearable technologies against reference tools is crucial to guarantee the accuracy and reliability of algorithms providing gait outcomes within specific populations (e.g., healthy, or clinical groups), enabling definitive performance and clinical decisions to be made [21, 22]. Following initial analytical validation within specific cohorts, wearable technology and outcomes can be examined for performance or clinical use in various settings (e.g., lab or outdoor/real-world), contributing to a deeper understanding of running gait in both clinical (e.g., neurological, musculoskeletal, or cardio-pulmonary conditions) [23] and sporting contexts (e.g., performance, fatigue, and injury mechanisms) [18].

The ViMove2 System (DorsaVi, www.dorsavi.com/vimove/) is a relatively low-cost commercially available wearable technology that comprises of tibia-based magnetometer inertial measurement units (MIMUs), which can provide the most reported temporal running gait outcomes (i.e., cadence and ground contact time (GCT)) [19]. However, despite being commercially available, the ViMove2 system has never had analytical validation performed, therefore the specific performance characteristics of the technology (i.e., error rates, accuracy etc.) are unknown. Therefore, this study aimed to; 1) determine the analytical validity of temporal running gait outcomes from the ViMove2 in health adults; and 2) examine the intra-session reliability of temporal running gait outcomes from the ViMove2 system in healthy adults. We hypothesized that ViMove2 would have good analytical validity and reliability of temporal running gait outcome measures.

## Methods

The following is a brief overview of the study methodology, for detailed information please see the published protocol; (ClinicalTrials.gov NCT05277181) [24].

### Participants

Ethical approval was granted by the Northumbria University Research Ethics Committee (Reference: 33358) and this study conformed to the Declaration of Helsinki. Recruitment took place from 02/01/2022 until 02/09/2022. All participants were supplied with informed consent and gave verbal and written consent before performing treadmill-based testing in Northumbria University’s Gait and Biomechanics Laboratory.

Adult participants with a range of running abilities were recruited from running clubs in the North-East of England. Inclusion criteria required participants to be aged 18 years or older, able to run unassisted for short periods. Prior to testing, all participants completed a questionnaire to provide information pertaining to their demographics, lifetime athletic injury history, medical history, sporting pursuits and running personal bests. Injury was classified as “any muscle, bone, tendon or ligament pain in the lower back/legs/knee/foot/ankle that caused a restriction or stoppage of running (distance, speed, duration or training) for at least 7 days or 3 consecutive scheduled training sessions, or that required the runner to consult a physician or other health professional” [25]. Exclusion criteria included medical history of disability that affects running gait safety or ability to follow instructions/tasks and any known illness or disease that would prevent their participation in strenuous physical activities (e.g., cardio-respiratory conditions or acute COVID-19).

### Instrumentation

#### Wearable technology: ViMove2

The ViMove2 consists of two MIMUs, each comprising a 3D accelerometer (±16g) and 3D gyroscope (±250 °/s) with a 3D magnetometer all sampling at 100Hz. MIMUs were placed onto the anterior surface of the mid-shank of the right and left tibia, as per manufacturer’s guidelines [26]. Tibia device placement is based on the anthropometric measurement of height and a ruler is used to measure exact placement, which is the midpoint between the knee and ankle along the anterior surface of the tibia [27]. After placement of bilateral tibia devices, the ViMove2 system was calibrated via its ViPerform software. Data were transmitted through wireless channel to a recording and feedback device, from which the data were subsequently offloaded onto a PC for further analysis.

#### Reference system: 3D motion capture system

The reference system consisted of a 14-camera 3D motion capture system, distributed around a space of 9.8 × 7.9 × 3.2m^3^, sampling at 250 Hz (VICON, Oxford, UK). The calibration of the Vicon system was conducted before each participant. Sixteen reflective markers were placed on the participants lower limb before testing, and a static calibration trial was initially collected to form a musculoskeletal model [28]. Using a small amount of double-sided tape, the markers were attached bilaterally to the following landmarks: anterior superior iliac spine, posterior superior iliac spine, mid-lateral thigh, lateral knee joint line, lateral mid-shank, lateral malleoli, calcaneal tuberosity, and base of the second metatarsal.

Participant specific information of weight, height, ankle width, knee width, and leg length (from posterior iliac spine to medial malleolus) were measured and inputted in the lower body model [29]. The Plug-in-Gait (PiG) lower body model was used to analyse movement at the joints and evaluate all parameters [30]. The lower body was modelled as seven segments (one pelvis, two thighs, two shanks, and two feet). A normal gait cycle was defined from the initial heel-to-heel contact with the same limb. Additional information of the PiG calculations can be found on Vicon’s website.

Data processing was performed in Vicon Nexus. All markers were labelled, and marker trajectories were filtered using a fourth order low-pass Butterworth filter via dynamic plug-in gait model with 6 Hz cut-off frequency. Gait identification was achieved through visual inspection of initial contact and toe off for consecutive strides over the trials. The trajectory of the heel and toe markers in the Z plane were examined, so that the minimum of the trajectory of one stride specified the timestamp of initial contact. The traced trajectory of the toe marker was used to specify the toe’s movement, so that the minimum of the trajectory specified the timestamp of a toe-off event. The initial contact and toe-off events of left and right foot steps were combined in order to estimate for each step GCT, and cadence over the duration of the trial [31]. Ground contact time and swing time were defined by the time between initial contact and toe-off events and between toe-off and initial contact events, respectively.

### Procedures

Prior to commencing the protocol, participants were provided the opportunity to run on the treadmill (Spirit fitness XT485) at a comfortable speed for a warm-up and to familiarise themselves. Participants were asked to run at five speeds, four of these speeds were standardised (i.e., 8, 10, 12 and 14 km/hr) and one speed was their self-selected speed. If a participant could not reach a certain speed (i.e., 12 or 14km/hr) or did not feel comfortable at that speed, then it was not completed. Self-selected speed was determined by the participant’s 5km personal best. The order of speed was consistent across participants, starting at the slowest speed (i.e., 8km/hr) and progressing to the fastest (i.e., 14 km/hr or 5km pace). Data were collected for 60 seconds at each speed. A period of 60s was chosen as it generally aligns with other similar studies in the field with data capture periods ranging from 20s [32] to 90s [19, 33, 34]. Participants could have breaks between trials or could abort the trial at any time. Participants were provided with a standardised, neutral cushioning running shoe (Saucony Guide Runner) to wear during testing to ensure consistency and remove bias from gait-affecting cushioning within e.g., support cushioning running shoes [35]. The ViMove2 system and 3D motion capture were recorded simultaneously to allow direct comparison of running gait outcomes. To assess intra-session reliability, participants completed the protocol in the same format in a repeated-measures design, within the session (i.e., two trials recorded at each speed).

The outcome measures were temporal running gait characteristics of ground contact time and cadence. Ground contact time was defined as the time elapsed (ms) between initial contact (where the foot first makes contact with the ground) and final/terminal contact (where the foot last leaves the ground). The GCT was averaged over the one-minute trials. Cadence was defined as the number of steps taken during a given time (one-minute trials).

### Statistical analyses

Data analysis was conducted in SPSS v27 (SPSS Inc., Chicago, IL, USA). Shapiro-Wilks tests indicated a normal distribution of all data (p<0.05). For all data analysis, a statistical significance was classed as p < 0.05. Intra-class correlations (*ICC*_(2,1)_) were defined as poor (<0.50), moderate (0.50–0.75), good (0.75–0.90) or excellent (>0.90) [36]. Analytical validity and intra-session reliability were conducted across the whole group, and broken into different sex sub-groups (males, females).

#### Analytical validity

Face validity was conducted by comparing descriptive data on temporal running gait outcomes across different running speeds. Concurrent validity was performed using ICC models that examined absolute agreement of temporal gait outcomes from the ViMove2 system and the reference 3D motion capture system. Mean error were calculated between the ViMove2 system and the reference 3D motion capture data for descriptive purposes and are observed as an accuracy metric in the outcomes. Limits of agreement (LoA) between outcomes were calculated and Bland-Altman plots were used to visualize [37].

#### Intra-session reliability

To determine the intra-session reliability of the ViMove2 system, Pearson’s correlation coefficients (r), ICCs_(2, 1)_ and LoA between the two testing time-points were calculated [38].

## Results

### Participants

A total of seventy-four participants completed the validity aspect of the study (43 males, 31 females; 39.1 ± 12.61 years; 1.71 ± 0.14m; 71.3 ± 15.6kg). Participants exhibited a range of running abilities (5km personal best; 23:21 ± 08:27). Seventy-one participants completed the intra-session reliability aspect (41 males, 30 females; 39.4 ± 12.6 years; 1.72 ± 0.14m; 71.3± 15.8kg; 5km personal best; 23:04 ± 08:29). Some data loss was experienced at 8km/hr due a technical issue with the ViMove2 system and a low-cut off threshold whereby the ViMove2 requires people to be running at 6km/hr for it to be detected as running, yet for many participants running at 8km/hr the ViMove2 system did not detect them as running. Additionally, some dropout during higher speeds was experienced (Table 1). For the validation aspect, a total of 578 trials across all speeds were examined. For intra-session reliability analysis, 270 trials across all speeds were examined.

**Table 1:** Summary of number of participants across all speeds.

Task	Outcome	Total n	Male n (%)	Female n (%)
8 km/hr	Validity	69	39 (56.5)	30 (43.5)
	Reliability	65	37 (56.9)	28 (43.1)
10 km/hr	Validity	74	43 (58.1)	31 (41.9)
	Reliability	71	41 (57.7)	30 (42.3)
12 km/hr	Validity	74	43 (58.1)	31 (41.9)
	Reliability	70	42 (60.0)	28 (40.0)
14 km/hr	Validity	72	41 (56.9)	31 (43.1)
	Reliability	64	39 (60.9)	25 (39.1)

### Ground contact time

**Fig 1:**
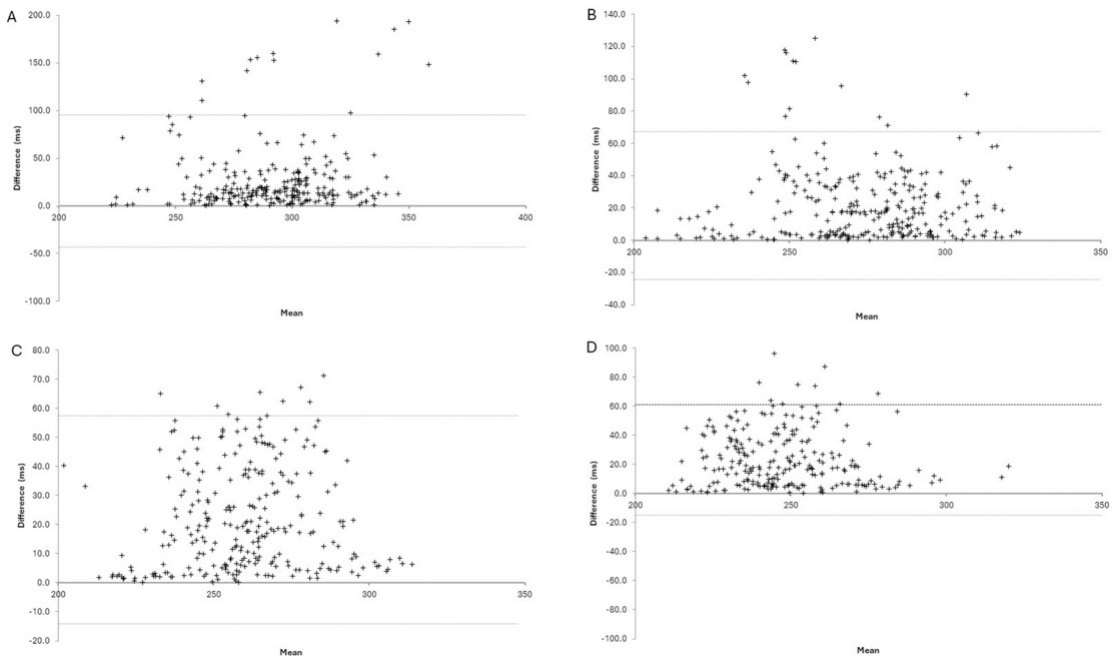
Bland-Altman plots illustrating the absolute agreement of GCT between the ViMove2 system and 3D motion capture system during treadmill running at various speeds. (A) 8km/hr, (B) 10 km/hr, (C) 12 km/hr, (D) 14km/hr.

**Fig 2:**
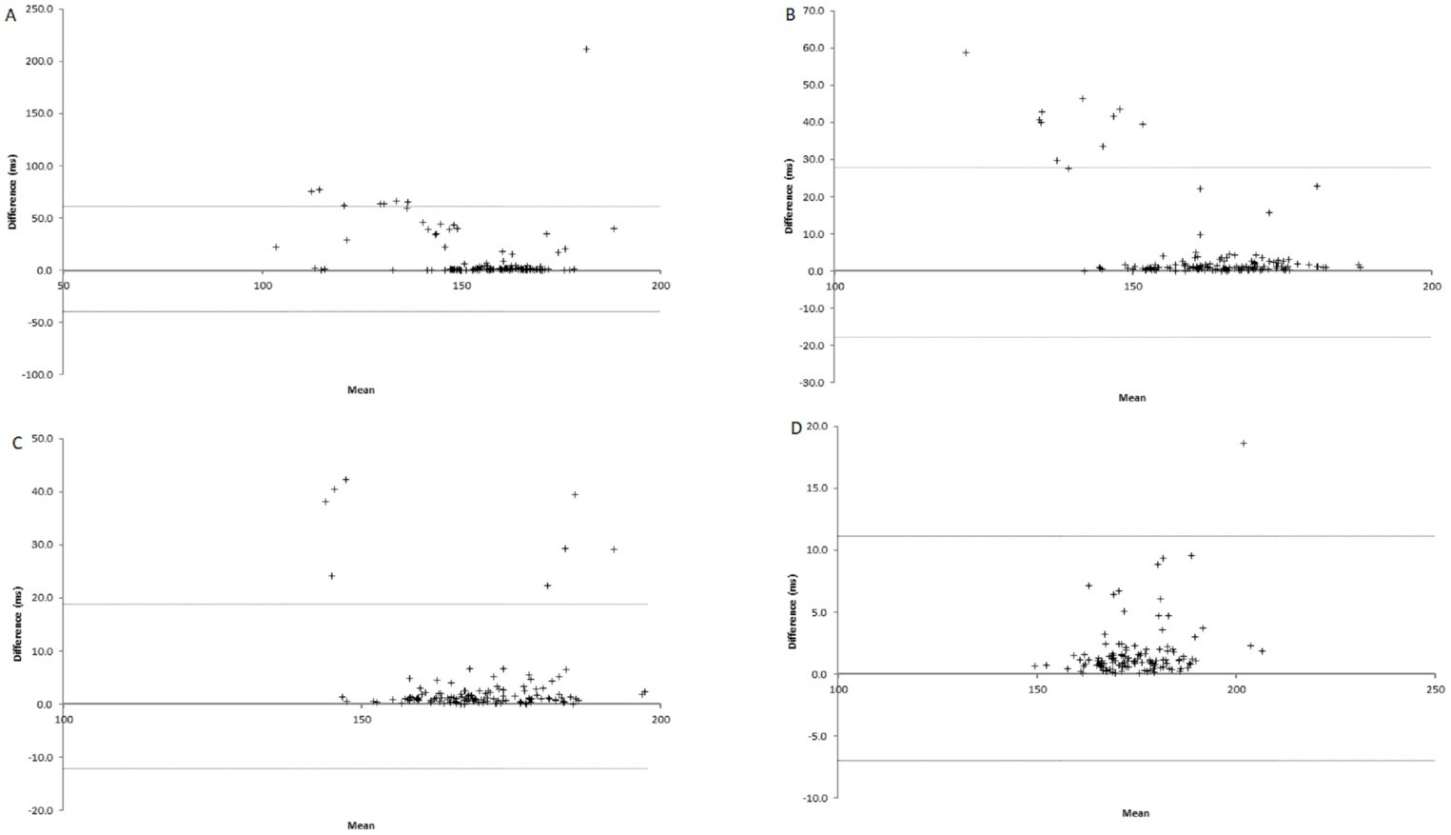
Bland-Altman plots illustrating the absolute agreement of cadence between the ViMove2 system and 3D motion capture system during treadmill running at various speeds. (A) 8km/hr, (B) 10 km/hr, (C) 12 km/hr, (D) 14km/hr.

Table 2 shows the descriptive gait data statistics from, along with the absolute agreement between the two systems for ICC, LoA (% and 95%) and r values. The agreement between the ViMove2 system and 3D motion capture is displayed in Figs 1 and 2.

**Table 2:** Mean difference, ICC(2,1), limits of agreement (LOA%), and Pearson correlation between the 3D motion capture reference and the ViMove2 system during running.

Task	Outcome	ViMove2	Reference System	Validity							
		Mean (SD)	Mean (SD)	Mean Difference	ICC	Lower Bound	Upper Bound	LoA (%)	LoA95%	*Pearson r*	*Pearson p*
**8 km/hr**	GCT (ms)	297.15 (37.53)	285.17 (27.29)	26.03	0.30	0.11	0.45	23.41	67.93	0.19	0.003
**(n = 69)**	Cadence (steps/min)	150.94 (21.98)	160.51 (16.87)	10.68	0.56	0.38	0.69	31.85	49.59	0.45	<0.001
**10 km/hr**	GCT (ms)	280.61 (31.25)	266.09 (25.68)	21.37	0.69	0.61	0.76	16.43	44.90	0.54	<0.001
**(n = 74)**	Cadence (steps/min)	160.76 (15.30)	164.37 (9.40)	4.93	0.72	0.61	0.80	13.74	22.33	0.63	<0.001
**12 km/hr**	GCT (ms)	296.57 (24.47)	249.04 (21.70)	21.60	0.79	0.74	0.84	13.54	35.12	0.66	<0.001
**(n = 74)**	Cadence (steps/min)	168.92 (11.41)	170.57 (10.64)	3.26	0.84	0.78	0.89	8.94	15.18	0.73	<0.001
**14 km/hr**	GCT (ms)	257.06 (22.70)	235.82 (22.80)	22.95	0.72	0.66	0.79	15.12	37.27	0.58	<0.001
**(n = 72)**	Cadence (steps/min)	174.32 (9.84)	174.74 (9.84)	2.06	0.93	0.90	0.95	5.08	8.87	0.87	<0.001

Face validity of GCT was shown via the decrease in time with increased running speed (Table 2 and Fig 1). The concurrent agreement for GCT from ViMove2 system and reference were weakest during running at 8km/hr, demonstrating poor agreement (ICC_(2,1)_ 0.30, LoA% 23.41). For running at 10km/hr and 14km/hr moderate agreement was displayed (ICC_(2,1)_ 0.69 and 0.72, LoA% 16.43 and 15.12, respectively) (Table 2 and Fig 1). Intraclass correlations show good agreement during running at 12km/hr (ICC_(2,1)_ 0.79, LoA% 13.54) (Table 2). GCT was slightly overestimated by the ViMove2 system compared to the reference system, with higher mean differences, particularly at slower speed (Table 2).

Concurrent validity of the ViMove2 system was impacted by sex. Females exhibited poor validity during 10, 12 and 14 km/hr trials (ICC_(2,1)_ 0.39, 0.44 and 0.42, LoA% 20.32, 14.00 and 15.78, respectively), whereas males had good to moderate validity for GCT during 10, 12 and 14 km/hr trials (ICC_(2,1)_ 0.88, 0.72 and 0.62, LoA% 12.52, 13.17 and 14.47, respectively) (S1 Table).

With respect to reliability, intraclass correlations showed good reliability for GCT at 8km/hr (ICC_(2,1)_ 0.82, LoA% 17.6), and excellent reliability (ICC_(2,1)_ >0.900, LoA% 6.13 to 6.56) and excellent reliability across all additional speeds (10, 12, and 14 km/hr) Table 3. Sex had no significant impact on the reliability of the ViMove2 system (S2 Table).

**Table 3:** Mean difference, ICC(2,1), limits of agreement (LOA%), and Pearson correlation between test and retest for the ViMove2 system during running.

Task	Outcome	Test	Retest	Reliability							
		Mean (SD)	Mean (SD)	Mean Difference	ICC	Lower Bound	Upper Bound	LoA (%)	LoA95%	*Pearson r*	*Pearson p*
**8 km/hr**	GCT (ms)	294.74 (37.43)	299.73 (27.72)	14.54	0.82	0.75	0.87	17.16	51.00	0.70	<0.001
**(n = 65)**	Cadence (steps/min)	150.68 (22.43)	150.92 (22.29)	5.32	0.93	0.89	0.96	12.86	19.39	0.88	<0.001
**10 km/hr**	GCT (ms)	280.40 (30.69)	280.57 (32.16)	8.02	0.96	0.95	0.97	6.34	17.66	0.93	<0.001
**(n = 71)**	Cadence (steps/min)	161.57 (14.34)	159.67 (16.37)	4.64	0.90	0.84	0.94	10.89	17.72	0.82	<0.001
**12 km/hr**	GCT (ms)	270.73 (24.40)	268.11 (24.59)	7.77	0.95	0.92	0.96	6.13	16.39	0.90	<0.001
**(n = 70)**	Cadence (steps/min)	168.20 (12.85)	170.21 (10.38)	3.16	0.90	0.84	0.94	10.74	18.17	0.84	<0.001
**14 km/hr**	GCT (ms)	256.55 (23.46)	257.56 (22.26)	7.18	0.94	0.91	0.96	6.56	16.85	0.88	<0.001
**(n = 64)**	Cadence (steps/min)	174.75 (9.14)	174.10 (10.48)	2.46	0.92	0.87	0.95	5.64	9.85	0.86	<0.001

### Cadence

Face validity of cadence measurement with the ViMove2 system was shown with the increased cadence with increased running speed (Table 2 and Fig 2). There was moderate agreement between the ViMove2 and reference system was shown when running at 8 km/hr (ICC_(2,1)_ 0.56, LoA% 31.85). The agreement between systems was good to excellent at speeds of 10 km/hr, 12 km/hr, and 14 km/hr (e.g., ICC_(2,1)_ 0.72, 0.84, and 0.93, respectively, Table 2 and Fig 2). Additionally, at slower speeds the ViMove2 system underestimated cadence compared to the reference system, as mean differences decreased in cadence decreased with increased speed (Table 2 and Fig 2).

Minor variations in concurrent validity were observed for measuring cadence in different sexes. Specifically, running at 14km/hr the females exhibited moderate validity for cadence (ICC_(2,1)_ 0.86, LoA% 8.01), whereas males had excellent validity (ICC_(2,1)_ 0.97, LoA% 2.58) (S1 Table).

Cadence demonstrated excellent reliability across all speeds (ICC_(2,1)_ >0.900, LoA% 5.64 to 12.86) Table 3. Sex did not significantly impact the reliability of the ViMove2 system when measuring cadence (S2 Table).

## Discussion

This is the first study to examine the analytical validity and reliability of temporal running gait parameters in healthy adults measured by the commercially available ViMove2 system. Overall, the findings indicated that the ViMove2 system is a valid digital health technology for measurement of GCT and cadence in healthy adults, exhibiting expected alterations in outcomes across different running speeds and being comparable to the established ’gold-standard’ reference technology (i.e., 3D motion capture). The ViMove2 system also demonstrated high levels of intra-session reliability across different speeds and trials. However, there are specific factors that influence the validity and reliability that need to be considered when interpreting results (e.g., speed of running and sex of participant), which are discussed further in this section. This study provides the performance characteristics of the ViMove2 system for temporal running gait outcomes, which can be used to determine whether future results are meaningful (e.g., differences in performance are beyond error (mean bias) in the outcome). Understanding the accuracy and reliability of running gait outcomes from relatively low-cost commercial wearable technology is crucial in providing specific data to determine error rate and will allow calculation of minimal important differences in future work [39], which is vital to implementation in applied settings and to reduce reliance on expert analysis and/or gold-standard, high-cost technologies [40].

### Ground contact time

Ground contact time is essential to understand due to the implications on running economy [41]. There was a consistent decrease in GCT with increased running speed across systems in this study, which aligns with previous research and demonstrates face validity of GCT measured by the ViMove2 system in healthy adults [42]. Furthermore, on average, GCT values recorded by the ViMove2 system were slightly higher than the reference system across all running speeds, which indicates a systematic overestimation of the GCT by the wearable system (Table 2 and Fig 1). The difference between the wearable and reference systems may relate to the inherent differences in the measurement technologies, with one system attached to the participant and the other relying on video capture (e.g., devices attached to the participant may be able to detect subtle changes in movement prior to the video capture).

In terms of validity, the ViMove2 system GCT had moderate agreement to the reference standard for speeds above 10km/hr but had poor agreement at 8km/hr. Similarly for reliability, the ViMove2 system had excellent intra-session reliability for speeds >10km/hr but only moderate reliability for running at 8km/hr. Validity and reliability of the ViMove2 system was generally better with faster running speed (Tables 2 and 3, Fig 1), which contrasts similar work in the field [31, 43]. For example, Falbriard et al., (2018) demonstrated that research-grade IMU GCT validity degrades with faster speed and our previous work with the high-end commercial wearable system from DANU Sport Ltd. demonstrated excellent validity and reliability (ICC_(2, 1)_ >0.90) for GCT across treadmill running speeds [31, 43]. The ViMove2 system is a commercial technology that has a proprietary (‘black-box’) algorithm for processing MIMU data, and therefore we are unable to determine the exact reason for inaccurate GCT at slower speeds. However, the lack of accuracy at slower speed likely relates to the underlying algorithm thresholds for initial and final contact being inaccurate, e.g., a lower-bound threshold for gait event detection is possibly impacting the temporal data when running at slower speeds. Indeed, the ViMove2 system instructions require runners to attain a minimum running speed threshold of 6km/hr for accurate detection and data capture.

Several of the participant data collection was lost due to the error of running too slowly (i.e., the system reported not being able to detect the participant), even though they were running at 8km/hr on the treadmill. Additionally, at slow speeds there may be extraneous noise due to inconsistent running patterns or movements that can lead to misidentification of initial or final contact events [42]. Regardless, results highlight the importance of considering running speed when interpreting GCT data from the ViMove2 system, as it should be considered when interpreting results, with a lack of accuracy at speeds of 8km/hr meaning that GCT should not be reported at this speed range (e.g., it may require further work to determine speed dependent correction of results).

GCT validity was different for female and male runners. Females exhibited poorer concurrent validity for GCT compared to males at several running speeds, indicating that the GCT measurement via the ViMove2 system may be less accurate in females (S1 Table). The influence of sex on concurrent validity may relate to the ViMove2 system data analysis algorithm not being able to accurately detect the initial and final foot contacts during running within females who inherently have different running gait patterns and muscle activation than male runners [44–46]. With the ViMove2 algorithm being proprietary we cannot fully understand the specific reason for the difference in validity for female runners, so further research is required to investigate and compare different algorithms to ensure fit-for-purpose and valid outcomes for this population.

Discrepancies in GCT may have occurred due to the running protocol, e.g., running on a treadmill running may have influenced timing of movement and landing patterns (i.e., initial and final contact events) particularly at higher speeds [47]. The use of treadmill running in a laboratory environment was necessary as an initial form of analytical validation (e.g., the beginning of any analytical validation requires an extremely controlled protocol and environment to ensure data quality), however, future research should consider validating the device in a more ecologically valid setting.

### Cadence

Cadence was shown to increase with faster running speed, which demonstrates face validity of the ViMove2 system for measuring this temporal running gait outcome. Additionally, cadence was measured in healthy adults by the ViMove2 system with moderate to excellent validity (Table 2 and Fig 2). Mean difference (error/bias) in cadence measured by the ViMove2 compared to the 3D motion capture system was low across running speeds (i.e., 2-11steps/min), this is in line with other commercial wearable technologies that reported errors of ≤1% for cadence [48, 49], although higher errors have also been reported [50]. Previous guidelines for step-related wearable metrics suggest that an error of <5% is needed for use in clinical trials, while a 10%–15% error may be acceptable for the general population [51], which indicates that ViMove2 may be appropriate for clinical use. Interestingly, there was a consistent improvement in ICCs and reduction in mean differences between the ViMove2 and reference system for cadence with increased running speed (Table 2), which implied better agreement between the two systems at higher running speeds. The observed improvements in ViMove2 validity for cadence with increased running speed is consistent with previous research, which indicates that gait dynamics at faster speeds tend to be more predictable and reproducible, facilitating improved synchronization and agreement between measurement systems [49, 52]. For example, at lower speeds, variations in step length and duration may contribute to discrepancies in cadence measurements between systems. However, as speed increases, stride length tends to shorten, resulting in more uniform step intervals and facilitating improved synchronization and agreement between the ViMove2 and reference system [53–55].

At higher speed (14km/hr), validity of the cadence measurement by ViMove2 was impacted by sex with females only having moderate concurrent validity but males having excellent validity, similar to GCT measurement (S1 Table). Cadence measurement with the ViMove2 system algorithm may be influenced by the specific running patterns of the female and male runners. Specifically, the algorithm may be reliant on the limited lower limb movement in frontal and transverse planes that males show compared to females when running [46, 56], as this may create noise within the signal that is not being controlled for. Further work is needed to understand how different running mechanics may influence algorithm and device validity, as well as how different algorithms may be deployed to overcome limitations.

Cadence measurements demonstrated excellent intra-session reliability across all running speeds, with percentage LoA ranging from 5.64% to 12.86%, suggesting strong agreement between test and retest measurements (Table 3). These findings collectively underscore the reliability of cadence measurements as collected by the ViMove2 system Facross a range of speeds.

### Study limitations

This study has several limitations that should be addressed in future research. The first limitation is the validation of running gait during treadmill running in a laboratory, which may influence performance (e.g., running metrics, psychological factors etc.) and may not represent validity in settings where the wearable technology will be used (e.g., outdoor uncontrolled environments). In future studies could perform outdoor validation through comparison to other reference standards (e.g., instrumented insoles, 3D motion capture or 2D video capture etc.), which could then be applied to a range of testing environments [57, 58]. The second limitation was that it is currently not possible for the ViMove2 system and the 3D motion capture system to be electronically synchronized for data collection, therefore offline timestamps were used to ensure that the same steps were included in all analysis. Finally, while the current study included a range of running speeds for validity and reliability testing there are a range of other factors that may be considered in future work. For example, different surfaces, gradients, and direction changes, as well as in clinical populations.

## Conclusions

This study investigated the analytical validity and reliability of a commercial low-cost wearable technology, ViMove2, for measurement of temporal running gait outcomes during treadmill running in healthy adults. Overall, the ViMove2 system can measure GCT and cadence with comparable accuracy to a technological reference standard, but accuracy and reliability is affected by running speed and the sex of the participant. The ViMove2 system has poor validity and moderate reliability at 8km/hr, which indicates that speed should be considered when interpreting findings and conducting research. The ViMove2 system also had poor to moderate validity for GCT and cadence respectively for female runners, which may relate to inherent sex-related running mechanic differences and requires further investigation into underlying proprietary algorithms to determine the exact cause. Future work is required to further examine validity of the ViMove2 in more ecologically valid ‘real-world’ settings and to establish minimal important differences for clinical or performance applications.

## Supporting information

S1 Table Mean difference, ICC(2,1), limits of agreement (LOA%), and Pearson correlation between the 3D motion capture reference and the ViMove2 system during running separated by sex. (DOCX)

S2 Table Mean difference, ICC(2,1), limits of agreement (LOA%), and Pearson correlation between test and retest for the ViMove2 system separated by sex. (DOCX)
