# Supplementary material for: Validity and reliability of running gait measurement with the ViMove2 system
Source: PLoS One. 2024 Oct 31;19(10):e0312952. doi: 10.1371/journal.pone.0312952 (PMC11527157; doi:10.1371/journal.pone.0312952)
Supplement: S1 Table — (DOCX) [file pone.0312952.s001.docx]

| Task |  | Outcome | ViMove2 | Reference System | Validity | | | | | | | |
| --- | --- | --- | --- | --- | --- | --- | --- | --- | --- | --- | --- | --- |
|  |  |  | Mean (SD) | Mean (SD) | Mean Difference | ICC | Lower Bound | Upper Bound | LoA (%) | LoA95% | *Pearson*  *r* | *Pearson* *p* |
| 8 km/hr | Male | GCT (ms) | 305.25 (35.14) | 287.85 (21.84) | 25.43 | 0.28 | 0.02 | 0.47 | 21.29 | 62.74 | 0.21 | 0.012 |
| (n = 69) | (n = 39) | Cadence (steps/min) | 146.90 (24.53) | 159.03 (20.91) | 13.05 | 0.50 | 0.20 | 0.69 | 39.69 | 60.77 | 0.44 | <0.001 |
|  | Female | GCT (ms) | 287.21 (38.30) | 281.84 (32.70) | 28.03 | 0.24 | 0.10 | 0.47 | 26.44 | 74.66 | 0.14 | 0.145 |
|  | (n = 30) | Cadence (steps/min) | 155.73 (17.45) | 162.59 (10.83) | 8.08 | 0.49 | 0.15 | 0.70 | 19.80 | 31.51 | 0.40 | 0.002 |
| 10 km/hr | Male | GCT (ms) | 284.34 (28.72) | 267.08 (25.70) | 17.90 | 0.88 | 0.83 | 0.91 | 12.52 | 34.53 | 0.78 | <0.001 |
| (n = 74) | (n = 43) | Cadence (steps/min) | 157.01 (16.40) | 161.53 (9.21) | 5.79 | 0.68 | 0.51 | 0.80 | 15.51 | 24.70 | 0.61 | <0.001 |
|  | Female | GCT (ms) | 275.49 (33.76) | 264.74 (25.58) | 26.32 | 0.39 | 0.13 | 0.58 | 20.32 | 54.89 | 0.25 | 0.005 |
|  | (n = 31) | Cadence (steps/min) | 165.93 (11.84) | 168.28 (8.17) | 3.74 | 0.70 | 0.49 | 0.82 | 11.08 | 18.52 | 0.57 | <0.001 |
| 12 km/hr | Male | GCT (ms) | 270.87 (26.39) | 251.78 (23.90) | 20.14 | 0.72 | 0.06 | 0.88 | 13.17 | 34.42 | 0.72 | <0.001 |
| (n = 74) | (n = 43) | Cadence (steps/min) | 166.14 (12.13) | 167.42 (9.00) | 3.13 | 0.83 | 0.73 | 0.89 | 9.11 | 15.19 | 0.74 | <0.001 |
|  | Female | GCT (ms) | 267.58 (21.07) | 244.86 (17.00) | 23.83 | 0.44 | 0.16 | 0.71 | 14.00 | 35.88 | 0.49 | <0.001 |
|  | (n = 31) | Cadence (steps/min) | 173.17 (8.63) | 175.38 (11.14) | 3.46 | 0.79 | 0.64 | 0.88 | 8.77 | 15.28 | 0.69 | <0.001 |
| 14 km/hr | Male | GCT (ms) | 258.43 (22.28) | 240.35 (24.35) | 20.93 | 0.62 | 0.12 | 0.81 | 14.47 | 36.09 | 0.59 | <0.001 |
| (n = 72) | (n = 41) | Cadence (steps/min) | 172.72 (8.86) | 172.56 (9.65) | 1.61 | 0.97 | 0.96 | 0.98 | 2.83 | 4.89 | 0.95 | <0.001 |
|  | Female | GCT (ms) | 254.99 (23.18) | 229.03 (18.24) | 25.98 | 0.43 | 0.20 | 0.76 | 15.78 | 38.22 | 0.58 | <0.001 |
|  | (n = 31) | Cadence (steps/min) | 176.75 (10.73) | 178.06 (9.17) | 2.75 | 0.87 | 0.76 | 0.92 | 7.15 | 12.68 | 0.77 | <0.001 |

**S1 Table.** Mean difference, ICC(2,1), limits of agreement (LOA%), and Pearson correlation between the 3D motion capture reference and the ViMove2 System during running separated by sex.
