# Supplementary material for: Validity and reliability of running gait measurement with the ViMove2 system
Source: PLoS One. 2024 Oct 31;19(10):e0312952. doi: 10.1371/journal.pone.0312952 (PMC11527157; doi:10.1371/journal.pone.0312952)
Supplement: S2 Table — (DOCX) [file pone.0312952.s002.docx]

| Task | Sex | Outcome | Test | Retest | Reliability | | | | | | | |
| --- | --- | --- | --- | --- | --- | --- | --- | --- | --- | --- | --- | --- |
|  |  |  | Mean (SD) | Mean (SD) | Mean Difference | ICC | Lower Bound | Upper Bound | LoA (%) | LoA95% | *Pearson*  *r* | *Pearson* *p* |
| 8 km/hr | Male | GCT (ms) | 303.03 (35.41) | 307.59 (34.65) | 16.04 | 0.73 | 0.57 | 0.83 | 18.32 | 55.94 | 0.58 | <0.001 |
| (n = 65) | (n = 37) | Cadence (steps/min) | 146.34 (25.04) | 147.54 (24.31) | 6.34 | 0.93 | 0.86 | 0.96 | 15.32 | 22.51 | 0.86 | <0.001 |
|  | Female | GCT (ms) | 284.44 (37.31) | 289.97 (39.07) | 12.68 | 0.88 | 0.80 | 0.93 | 15.43 | 44.32 | 0.74 | <0.001 |
|  | (n = 28) | Cadence (steps/min) | 156.26 (16.98) | 155.26 (18.50) | 4.01 | 0.94 | 0.88 | 0.97 | 9.26 | 14.42 | 0.90 | <0.001 |
| 10 km/hr | Male | GCT (ms) | 283.71 (28.16) | 285.29 (29.79) | 8.00 | 0.96 | 0.94 | 0.97 | 5.92 | 16.66 | 0.92 | <0.001 |
| (n = 71) | (n = 41) | Cadence (steps/min) | 158.70 (14.54) | 154.71 (17.98) | 5.25 | 0.89 | 0.79 | 0.94 | 12.87 | 20.58 | 0.82 | <0.001 |
|  | Female | GCT (ms) | 275.87 (33.32) | 273.99 (34.07) | 8.04 | 0.97 | 0.94 | 0.98 | 6.94 | 18.81 | 0.93 | <0.001 |
|  | (n = 30) | Cadence (steps/min) | 165.50 (13.07) | 166.44 (10.64) | 2.12 | 0.91 | 0.80 | 0.96 | 8.08 | 13.41 | 0.85 | <0.001 |
| 12 km/hr | Male | GCT (ms) | 272.20 (25.79) | 269.49 (27.15) | 8.63 | 0.94 | 0.91 | 0.96 | 7.07 | 18.95 | 0.87 | <0.001 |
| (n = 70) | (n = 42) | Cadence (steps/min) | 164.81 (13.98) | 167.28 (9.22) | 4.38 | 0.85 | 0.72 | 0.92 | 12.22 | 20.29 | 0.78 | <0.001 |
|  | Female | GCT (ms) | 268.51 (21.94) | 266.02 (19.92) | 6.46 | 0.96 | 0.93 | 0.98 | 4.12 | 10.82 | 0.93 | <0.001 |
|  | (n = 28) | Cadence (steps/min) | 173.34 (8.68) | 174.65 (10.47) | 2.92 | 0.99 | 0.98 | 0.99 | 8.38 | 14.58 | 0.99 | <0.001 |
| 14 km/hr | Male | GCT (ms) | 258.30 (22.68) | 258.67 (22.42) | 6.61 | 0.95 | 0.92 | 0.97 | 5.66 | 14.62 | 0.90 | <0.001 |
| (n = 64) | (n = 39) | Cadence (steps/min) | 172.90 (8.62) | 172.72 (8.99) | 1.78 | 0.97 | 0.95 | 0.99 | 2.58 | 4.47 | 0.95 | <0.001 |
|  | Female | GCT (ms) | 254.03 (24.33) | 255.96 (21.92) | 8.01 | 0.92 | 0.86 | 0.95 | 7.65 | 19.50 | 0.86 | <0.001 |
|  | (n = 25) | Cadence (steps/min) | 177.41 (9.21) | 176.09 (12.03) | 2.71 | 0.86 | 0.69 | 0.94 | 8.01 | 14.15 | 0.79 | <0.001 |

**S2 Table.** Mean difference, ICC(2,1), limits of agreement (LOA%), and Pearson correlation between test and retest for the ViMove2 System separated by sex.
